# Poisoning by Antipyrin

**Published:** 1890-02

**Authors:** E. W. Berridge

**Affiliations:** London, England


### POISONING BY ANTIPYRIN.

E. W. Berridge, M. D., London, England.

June 14th, 1889, 6 P. M.—The Countess of —— took ten grains of Burrowes & Wellcome's *Antipyrin* for headache. She is sensitive to medicines, and had had much mental anxiety lately. She had been under my treatment for some time with great benefit; but as she called to-day after office hours, she did not find me at home, and so some "d——d good-natured friend," as the poet profanely says, persuaded her to take this drug, stating that he always cured his wife's headache with it.

(N. B.—The expression "always cured" shows that the "cure" was not permanent.)

In five minutes my patient was seized with tingling burning sensation round upper part of mouth, inside nostrils, and in palate, just like cayenne pepper ; this was immediately followed by ineffectual desire to sneeze ; then clear water poured from both nostrils. These symptoms ceased as suddenly as they had begun ; and were immediately followed by sensation of dryness and burning in left side of throat, with instantaneous swelling in left throat, and for a few moments absolute loss of voice. The aphonia soon gave way to hoarseness, with much coughing and expectoration which seemed to come from back of throat and nose. With these symptoms there was a lump about an inch thick in left cheek, just below level of upper teeth. Head perfectly clear, but great nervous anxiety, trembling of limbs and weakness in walking. These symptoms continued till 10 P. M., when the swelling in throat was relieved by sleep and hot drinks, but the hoarseness was unchanged.

At 3 A. M., after further sleep, the swelling in throat had almost entirely gone, but the same burning, tingling sensation was felt in vagina, also nervous pains all over body. She now also had two fainting spells, with sensation of the heart stopping ; throbbing all over body, coldness of hands and feet, and nervous shuddering without chill. Then, after taking half a wine-glass of brandy and water, she slept again, and at 7 A. M. next day all the symptoms had gone except the hoarseness, with weakness. The lump in the mouth passed away in half an hour. During the first symptoms, the left corner of mouth showed a tendency to draw down.

June 15th.—The hoarseness continues with fainting. Frequent passage of clear urine, much and often, all the time. Had a little delirium during sleep last night, seeing faces. Tongue white. Yesterday she looked at her throat in a mirror, and found it white, with left tonsil inflamed and swollen.

*Belladona*$^{200}$ soon removed the remaining symptoms, though the weakness persisted for some days.

This *Antipyrin* is the new drug which the allopaths are using indiscriminately because they have no law ; and the mongrels because they do not really believe in the law.

